# Rescuing Newcastle disease virus with tag for screening viral-host interacting proteins based on highly efficient reverse genetics

**DOI:** 10.3389/fvets.2024.1418760

**Published:** 2024-07-19

**Authors:** Ruiwei Wang, Xuhong Cao, Kejia Lu, Zhengwu Chang, Xiaoyu Dong, Hanwei Guo, Xi Wei, Ruyi Dang, Juan Wang, Xinglong Wang, Sa Xiao, Haijin Liu, Zengqi Yang

**Affiliations:** College of Veterinary Medicine, Northwest A&F University, Shaanxi Yangling, Xianyang, China

**Keywords:** Newcastle disease virus, reverse genetics, protein tag, M protein, viral-host interaction

## Abstract

The interaction between viral proteins and host proteins plays a crucial role in the process of virus infecting cells. Tags such as HA, His, and Flag do not interfere with the function of fusion proteins and are commonly used to study protein–protein interactions. Adding these tags to viral proteins will address the challenge of the lack of antibodies for screening host proteins that interact with viral proteins during infection. Obtaining viruses with tagged fusion proteins is crucial. This study established a new reverse genetic system with T7 promoter and three plasmids, which efficiently rescued Newcastle disease virus (NDV) regardless of its ability to replicate in cells. Subsequently, using this system, NDV containing a HA-tagged structural protein and NDV carrying a unique tag on each structural protein were successfully rescued. These tagged viruses replicated normally and exhibited genetic stability. Based on tag antibodies, every NDV structural protein was readily detected and showed correct subcellular localization in infected cells. After infecting cells with NDV carrying HA-tagged M protein, several proteins interacting with the M protein during the infection process were screened using HA tag antibodies. The establishment of this system laid the foundation for comprehensive exploration of the interaction between NDV proteins and host proteins.

## 1 Introduction

As obligate intracellular parasites, viruses and their coevolved hosts have established intricate and dynamic interactions (1). Viruses manipulate host cellular processes for their replication and dissemination, triggering significant alterations in host cells, including changes in protein abundances, subcellular localizations, posttranslational modifications and secretion. In response to infection, hosts have evolved a series of intrinsic defense mechanisms to counter immediate viral replication (2). Understanding viral-host interactions can yield valuable insights into the biological functions of viral proteins, highlight interactions critical for the virus life cycle, and advance our understanding of viral pathogenicity, reproduction, and potential treatments. Newcastle disease virus (NDV) poses a significant threat to poultry (3). Moreover, NDV is also commonly used to alter the tumor immune microenvironment for cancer therapy. Therefore, understanding the interaction between Newcastle disease virus and host proteins during the process of infection is of great significance for the prevention and control of Newcastle disease and cancer treatment. However, the lack of commercialized antibodies against viral proteins has limited the study of interactions between NDV proteins and host proteins.

The reverse genetic technology has made it possible to modify viruses at the genetic level. In theory, it is feasible to fuse some commonly used tags such as HA, His, Flag, etc., to viral proteins to get the relevant viruses. This enables the use of tag antibodies to identify viral proteins during the viral infection, thus screening for proteins that interact with viral proteins. NDV belongs to non-segmented single-stranded negative-sense (NSNSV) virus, which requires a viral RNA-dependent RNA polymerase (RdRp) complex to replicate and transcribe nucleocapsid (N) protein-encapsidated viral RNA (4). Viral reverse genetics mimic mechanisms of viral RNA synthesis to rescue the virus. Therefore, the strategies of reverse genetics for NSNS viruses are quite similar (5). In 1994, the processes of developing reverse genetics of NSNS viruses (RV), including: (1) constructing RV full-genome, nucleoprotein (N), phosphoprotein (P), and large (L) protein expression plasmids; (2) transfecting plasmids into cells expressing T7 RNA polymerase; (3) amplifying rescued virus, were first reported (6). Over the last 20 years, various NSNS viruses have been generated using this method with some modifications. For instance, the In-Fusion method was employed to assemble viral full-genomes and required less time than the canonical digestion and ligation method (7). The number of plasmids transfected into cells was reduced by cloning the N gene and genes of RdRp into the same plasmid, generating cells persistently expressing RdRp or introducing the T7 promoter directly in front of genes of RdRp during the construction of the full-genome vector (8–12). The T7 RNA polymerase promoter was replaced by eukaryotic RNA polymerase promoters, such as CMV, which makes NSNS virus rescue independent of T7 RNA polymerase (9, 13). Regardless of the methods used, the quantity and quality of plasmids are crucial for the successful transcription of viral full-genomes (larger than 10 kb) by RNA polymerase *in vivo*, which is necessary for rescuing (5). Thus, plasmids harboring the full-genome of NSNS viruses were usually purified via Maxi prep. However, compared to Mini prep, Maxi prep is costly, time-consuming and complex for bench work.

In recent decades, there have been significant technological advances in proteomics approaches for investigating virology (14). One of the most commonly used methods is Affinity Purification-Mass Spectrometry (AP-MS), which has been successfully implemented to isolate virus-host protein complexes from infected cells using antibodies against the target viral protein. Traditional studies of virus-host interactions rely heavily on antibodies targeting the viral protein, which greatly limits the study of viral proteins due to the lack of specific antibodies. To overcome this limitation, AP has been put into effect by utilizing epitope-tagged viral strains (15). The acquisition of epitope-tagged viral strains through reverse genetic systems offers a novel tool for studying virus-host interactions in virally infected cells, as well as for protein tracing in live cells. However, several technical challenges must be taken into consideration when designing epitope-tagged viruses (2, 16), as the size and location of a tag do not affect virus growth or the function of the protein.

Here, we have successfully developed an efficient reverse genetics system for NDV based on Mini prep plasmids. This system operated under the T7 promoter. The additional T7 RNA polymerase was provided by a plasmid. Furthermore, this system included a plasmid expressing NP, P, and L proteins. Using this system, we rescued NDVs with tag on each structural protein and found that the tag did not alter the viral replication and genetic stability. By employing tag-specific antibodies, 100 host proteins and 5 viral proteins that interact with the viral M protein were identified during the viral infection.

## 2 Materials and methods

### 2.1 Cells and virus

BHK-21 cells were cultured in MEM Alpha (Gibco, 12,561), DF-1 and 293-T cells were cultured in DEME (Gibco, 2,898,684) supplemented with 10% fetal bovine serum (FBS, Gibco, 10,099) at 37°C with 5% CO_2_. Competent *E. coli*, NEB 10-beta strain, was acquired from NEB. NDV LaSota and C22 strains were propagated in 9-day-old chicken embryos and stored at −80°C.

### 2.2 Plasmid constructions

The complete genome of the NDV LaSota strain with the mCherry gene was assembled into the pCMV vector to create pCMV-LaSota/Cherry, as previously described (9). The F gene of LaSota was modified to include the velogenic cleavage site (Fmu) using overlap PCR. Subsequently, the modified F gene was substituted for the F gene in pCMV-LaSota/Cherry, resulting in the generation of pCMV-LaSota/Cherry/Fmu. The CMV promoter in both pCMV-LaSota/Cherry and pCMV-LaSota/Cherry/Fmu was replaced with the T7 promoter, yielding pT7-LaSota/Cherry and pT7-LaSota/Cherry/Fmu. Next, the mCherry gene in these vectors was replaced with either GFP or BFP genes, leading to the generation of pT7-LaSota/GFP, pT7-LaSota/BFP, pT7-LaSota/GFP/Fmu, and pT7-LaSota/BFP/Fmu plasmids. The pT7-C22 vector was engineered by incorporating HA tag within C-terminus of NP, M, F, HN, and L proteins, respectively and an HA tag was inserted within the N-terminus of the P protein using RT-PCR with specific primers containing HA tag, resulting in pT7-C22-NP-HA, pT7-C22-P-HA, pT7-C22-M-HA, pT7-C22-F-HA, pT7-C22-HN-HA, and pT7-C22-L/-HA. Additionally, the VSV, His, Flag, HA, and Myc tags were fused at the C-terminus of NP, M, F, HN, and L proteins, respectively, and the V5 tag was inserted at the N-terminus of the P protein, generating pT7-C22-NP/VSV-P/V5-M/His-F/Flag-HN/HAHA-L/Myc. A mini-genome containing the NDV leader, GFP, and NDV trailer was inserted in reverse orientation under the CMV promoter to produce pCMV-Mini. Subsequently, the CMV promoter was replaced with T7 to obtain pT7-Mini. The NP, P, and L genes of LaSota were individually amplified by RT-PCR and cloned into pCI-neo or pBluescript SK vectors to generate pCMV-NP, pCMV-P, pCMV-L, pT7-NP, pT7-P, and pT7-L plasmids. These NP, P, and L cassettes, each containing the CMV promoter and polyA sequence, were sequentially cloned into the same pCMV vector to generate pCMV-NP-P-L plasmids, as previously reported (9). The T7 polymerase gene was amplified from BL-21 *E. coli* and cloned into the pCAGGS vector to create pCAGGS-T7. The M gene of C22 with an HA tag, ACTG1 and RPL4 genes with Flag tags were amplified by RT-PCR and cloned into pCAGGS vectors to produce pCAGGS-M-HA, pCAGGS-ACTG1-Flag, and pCAGGS-RPL4-Flag.

All these plasmids were transformed into NEB 10-beta competent *E. coli* and purified using Mini prep (TIANGEN, China) or Maxi prep (QIAGEN) kits and confirmed by sequencing. Detailed information about these plasmids was provided in Supplementary Table S1.

### 2.3 Virus rescue

NDVs were rescued as described previously (9). Briefly, 4 × 10^5^ BHK-21 cells were seeded in one well of 6-well plates 1 day prior to transfection. The viral full-length plasmids under the CMV promoter (2.5 μg) and pCMV-NP-P-L (2.5 μg), or viral full-length plasmids under the T7 promoter (2 μg), pCMV-NP-P-L (2 μg), and pCAGGS-T7 (1 μg) were mixed with 10 μL of Lipofectamine 3000 (ThermoFisher) and transfected into the cells. Subsequently, cytopathic effects (CPE) and expression of fluorescent proteins were examined using a microscope. Three days post-transfection, cells and supernatants were collected and inoculated into the allantoic cavity of 9-day-old embryonated chicken eggs. The eggs were then incubated at 37°C for 3 days, followed by overnight cooling at 4°C before collecting the allantoic fluid. The presence of rescued NDV was confirmed by hemagglutination (HA) assay and sequencing.

### 2.4 Virus titration

The viruses in the supernatants of transfected cells were titered using TCID_50_. Briefly, 1 × 10^4^ BHK-21 cells were seeded in 96-well plates 1 day before titration. On the day of titration, the cells were washed with phosphate-buffered saline (PBS), and 100 μL of ten-fold diluted collected supernatants were added. The cells were then incubated for 1 h with shaking every 15 min. After incubation, the liquids were discarded and 100 μL of MEM containing 2% FBS was added. Five days after infection, viral titers were calculated based on fluorescence or CPE.

### 2.5 Mini-genome rescue

1 × 10^5^ BHK-21 cells were seeded in one well of 24-well plates 1 day before infection. A total of 1.2 μg of plasmids was directly transfected into the cells using 2.4 μL of TurboFect (ThermoFisher). The first group consisted of 0.3 μg of pCMV-Mini, 0.12 μg of pCMV-NP, 0.12 μg of pCMV-P, 0.08 μg of pCMV-L, and 0.6 μg of pCAGGS. The second group included 0.3 μg of pT7-Mini, 0.12 μg of pT7-NP, 0.12 μg of pT7-P, 0.08 μg of pT7-L, and 0.6 μg of pCAGGS-T7. The third group contained 0.3 μg of pCMV-Mini, 0.12 μg of pT7-NP, 0.12 μg of pT7-P, 0.08 μg of pT7-L, and 0.6 μg of pCAGGS-T7. The fourth group included 0.3 μg of pT7-Mini, 0.12 μg of pCMV-NP, 0.12 μg of pCMV-P, 0.08 μg of pCMV-L, and 0.6 μg of pCAGGS-T7. The last group comprised 0.3 μg of pT7-Mini, 0.3 μg of pCMV-NP-P-L, and 0.6 μg of pCAGGS-T7. Following transfection, GFP expression was monitored under a microscope every 12 h for 36 h. Additionally, at the 24th h post-transfection, cells were collected to determine the ratio of GFP-positive cells using flow cytometry.

### 2.6 Indirect immunofluorescence assay (IFA)

0.5 × 10^5^ BHK-21 cells were seeded in one well of 48-well plates 1 day before infection. After washing with PBS, the cells were infected with 1 MOI rescued NDV. At the 24th h post-infection, the cell supernatants were removed, and the cells were washed with PBS. The cells were then fixed with 4% paraformaldehyde (PFA) for 20 min at room temperature (RT). After discarding the PFA, the cells were washed and permeabilized with 0.2% Triton X-100 for 5 min at RT. Subsequently, the Triton X-100 was removed, and the cells were washed and blocked in PBS containing 1% bovine serum albumin (BSA) at 37°C for 30 min. The BSA was then removed, and 100 μL of 1% BSA containing antibodies against NDV HN, Tag VSV, V5, His, Flag, HA, or Myc (Cell Signaling Technology) were added to the wells. The plate was then incubated at 37°C for 2 h. After incubation, the primary antibodies were removed, and the cells were washed 5 times with PBS before being incubated with goat anti-mouse Alexa Fluor 488 or Alexa Fluor 599 secondary antibodies (Abcam) for 1 h at 37°C. Following this, the secondary antibodies were removed, and the cells were washed 5 times with PBS, incubated with Hoechst 33342 (Thermo Fisher) for 15 min at RT, and then imaged using a fluorescent microscope.

### 2.7 Western blot

1.2 × 10^6^ DF-1 cells were seeded in one well of 6-well plates 1 day before infection. After washing with PBS, the cells were infected with 0.01 MOI rescued NDV. At the 24 h post-infection, the cell supernatants were discarded and the cells were rinsed with PBS. Following the extraction and lysis of total protein from DF-1 cells in RIPA Lysis buffer, the protein was electrophoresed on 10% SDS-PAGE and then electroblotted onto polyvinylidene difluoride (PVDF) membranes. The membranes were blocked in 10% skimmed milk dissolved in 0.05% Tris-buffered saline containing 0.05% Tween 20 (TBST) for 2 h at room temperature, washed 3 times in 0.05% TBST, and incubated with primary antibodies overnight at 4°C. The primary antibodies were used to probe specific proteins at the following dilutions: anti-Tubulin (1:3000), anti-NP (1:1000), anti-Flag (1:1000), anti-HA (1:1000), anti-V5 (1:1000), anti-Myc (1:1000), anti-VSV (1:1000) and anti-His (1,1,000). The next day, the primary antibodies were removed, and the membranes were washed 3 times in TBST before being incubated with HRP-conjugated goat anti-mouse IgG (1,3,000) antibodies for 1 h at room temperature. After removing the secondary antibodies, the membranes were washed 3 times in TBST, and the proteins were detected with ECL supersensitive kit under the chemiluminescence imager.

### 2.8 Virus growth curve

6 × 10^5^ DF-1cells were seeded in one well of 12-well plates 1 day prior to infection. On the day of infection, the cells were washed with PBS and then infected with tag-labeled NDV C22 virus and the parent NDV C22 virus at 0.01 MOI. After absorption at 37°C for 1 h, unbound virus was rinsed with PBS twice, followed by the addition of 1 mL DMEM containing 2% FBS. The plates were then incubated in a 37°C incubator with 5% CO_2_ for 3 days. At specific time points (12, 24, 36, 48, 60, and 72 hpi), a small amount of medium (100 μL per well) was collected from each well, and the virus titer was determined by TCID_50_, as described previously.

### 2.9 Immunoaffinity purification and mass spectrometry analyses

The DF-1 cells were infected with rC22-M-HA and the parent NDV C22 virus at 0.1 MOI. At the 24 h after infection, the cells were washed with PBS and lysed in RIPA buffer containing a 1/200 (v/v) protease inhibitor mixture. Following centrifugation, the supernatant was transferred to new tubes and then incubated with anti-HA antibody for 1 h at 4°C, and coupled to Protein A/G PLUS-Agarose overnight at 4°C. After resuspending the collected immune precipitates in 1x electrophoresis sample buffer, the samples underwent a 10-min boiling period and were subjected to SDS-PAGE analysis. Each protein sample’s gel lane (between 35 kDa to 50 KDa) was cut into 1-millimeter pieces, subjected to trypsin digestion, and then analyzed using mass spectrometry by Shang hai Bioprofiletechnology, China.

### 2.10 Immunoprecipitation

The 293-T cells were transfected with pCAGGS-M-HA and pCAGGS-ACTG1-Flag or pCAGGS-RPL4-Flag plasmids, and with pCAGGS and pCAGGS-ACTG1-Flag or pCAGGS-RPL4-Flag plasmids as a control. At the 24th h after transfection, the cells were washed with PBS and lysed in RIPA buffer containing protease inhibitor. After centrifugation, the supernatant was transferred to new tubes. A small portion of the supernatant was kept as input sample, and the remaining supernatant was incubated with anti-HA antibody for 1 h at 4°C. Protein A/G PLUS-Agarose (10 μL) was added, and the mixture was incubated at 4°C on a rocker platform overnight. Immunoprecipitates were collected by centrifugation, and the pellet was washed four times with cold PBS. After the final wash, the supernatant was aspirated and discarded, and the pellet was resuspended in 20 μL of 1x electrophoresis sample buffer. Samples were boiled for 10 min and analyzed by Western blotting with HA and Flag antibodies.

### 2.11 Statistical analysis

Statistical analysis was performed using GraphPad Prism 9 software (GraphPad Software, Inc., La Jolla, CA, United States). The datas were were expressed as means ± standard deviations. Significance were defined with the one-way analysis of variance or two-tailed independent (^∗^*p* < 0.05, ^∗∗^*p* < 0.01).

## 3 Results

### 3.1 The CMV two-plasmid system failed to rescue NDV using Mini prep plasmids

**Figure 1:**
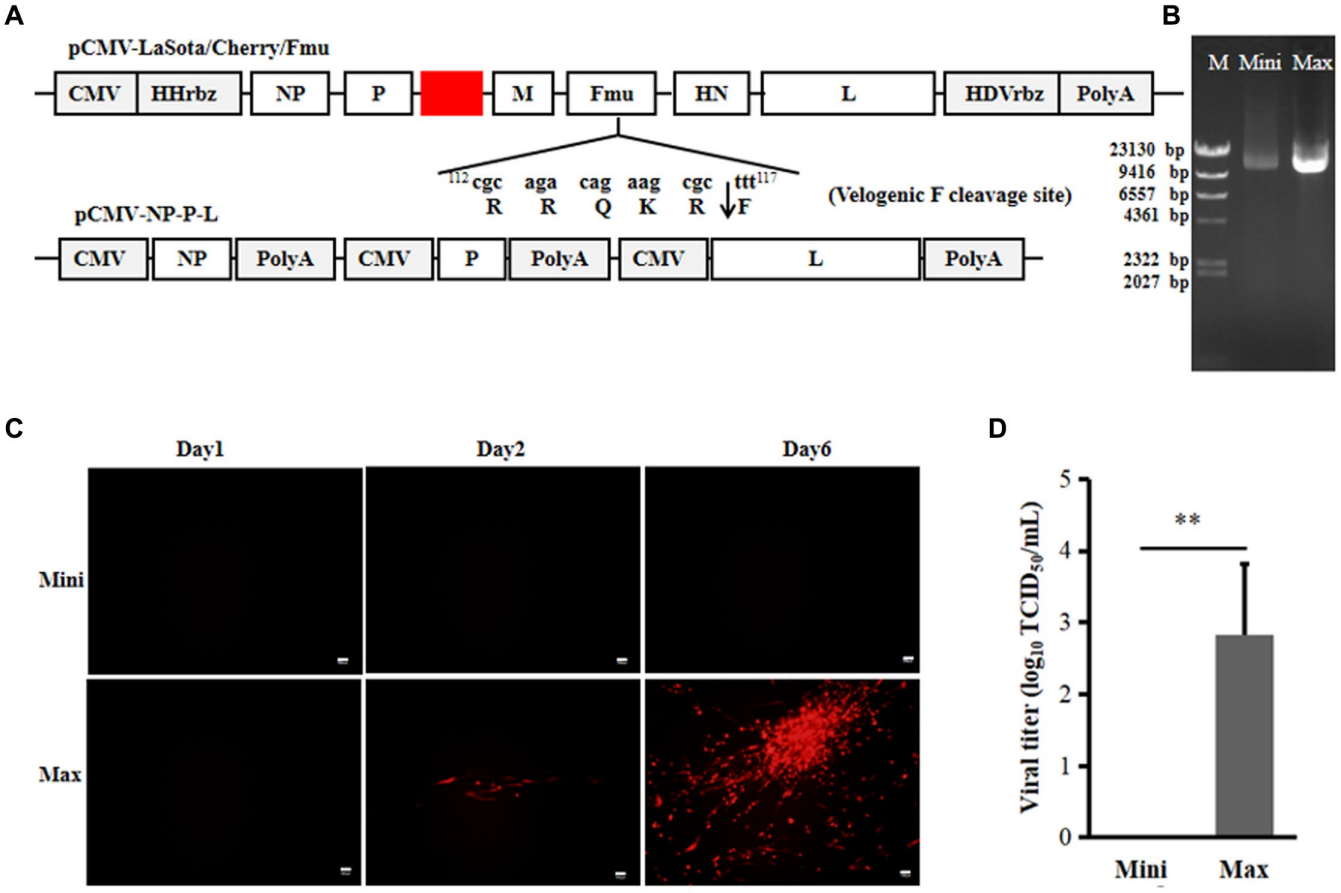
Efficiency of the 2-plasmid CMV NDV reverse genetics system. **(A)** Schematic representations of the pCMV-LaSota/Cherry/Fmu and pCMV-NP-P-L plasmids. The mCherry gene was depicted by the red rectangle. **(B)** Mini prep and Maxi prep of pCMV-LaSota/Cherry/Fmu plasmids. Electrophoresis was performed on a 0.8% agarose gel with 1 μg of plasmids loaded. M, Mini, and Max represent Marker, Mini prep, and Maxi prep, respectively. **(C)** Fluorescence expression in transfected cells. RFP expression was monitored daily for six days post-transfection. **(D)** Titration of rescued rLaSota/Cherry/Fmu. Supernatants from transfected cells were collected at the 6th d post-transfection, and NDV titers were determined on BHK-21 cells based on red fluorescence. ^**^p < 0.01.

To test the rescue of NDV from plasmids obtained via Mini prep, the NDV two-plasmid reverse genetics system was utilized (9). In this system, the full-length NDV LaSota strain was cloned under the CMV promoter in one plasmid. The NP, P, and L genes with independent CMV promoters and polyA sequences were inserted into another plasmid. This system efficiently rescued velogenic or lentogenic strains from plasmids prepared using Maxi prep. Additionally, the cleavage site of the viral F protein was modified from lentogenic-like to velogenic-like, and the mCherry gene was inserted between P and M, enabling visualization of the rescuing process (Figure 1A). Using this system, only rLaSota/Cherry/Fmu was generated from full-genome plasmids purified by Maxi prep. Two days post-transfection, red fluorescent cells were observed under the microscope. Over time, more cells expressed the cherry protein. Furthermore, cytopathic effects (CPE) became evident later in the transfection process (Figure 1C). Six days post-transfection, the NDV titer in the supernatants was around 10^3^ TCID_50_/ml (Figure 1D). In contrast, no red fluorescence or CPE appeared in cells transfected with full-genome plasmids purified by Mini prep (Figure 1C). Moreover, viral particles could not be detected in the supernatant (Figure 1D). Even when the transfected cells with supernatant were injected into chicken embryos, virus rescue was unsuccessful (data not shown). The 1 μg full-genome plasmids (plasmid concentration confirmed via Nanodrop) were loaded onto a gel for analysis. As shown in Figure 1B, in terms of quantity and quality, the Maxi prep plasmid was superior to the Mini-prep plasmid (Figure 1B). These results suggest that the poor quality of the Mini prep full-genome plasmid may impact NDV rescue.

### 3.2 The T7 NDV reverse genetics demonstrated high efficiency at the mini-genome level

**Figure 2:**
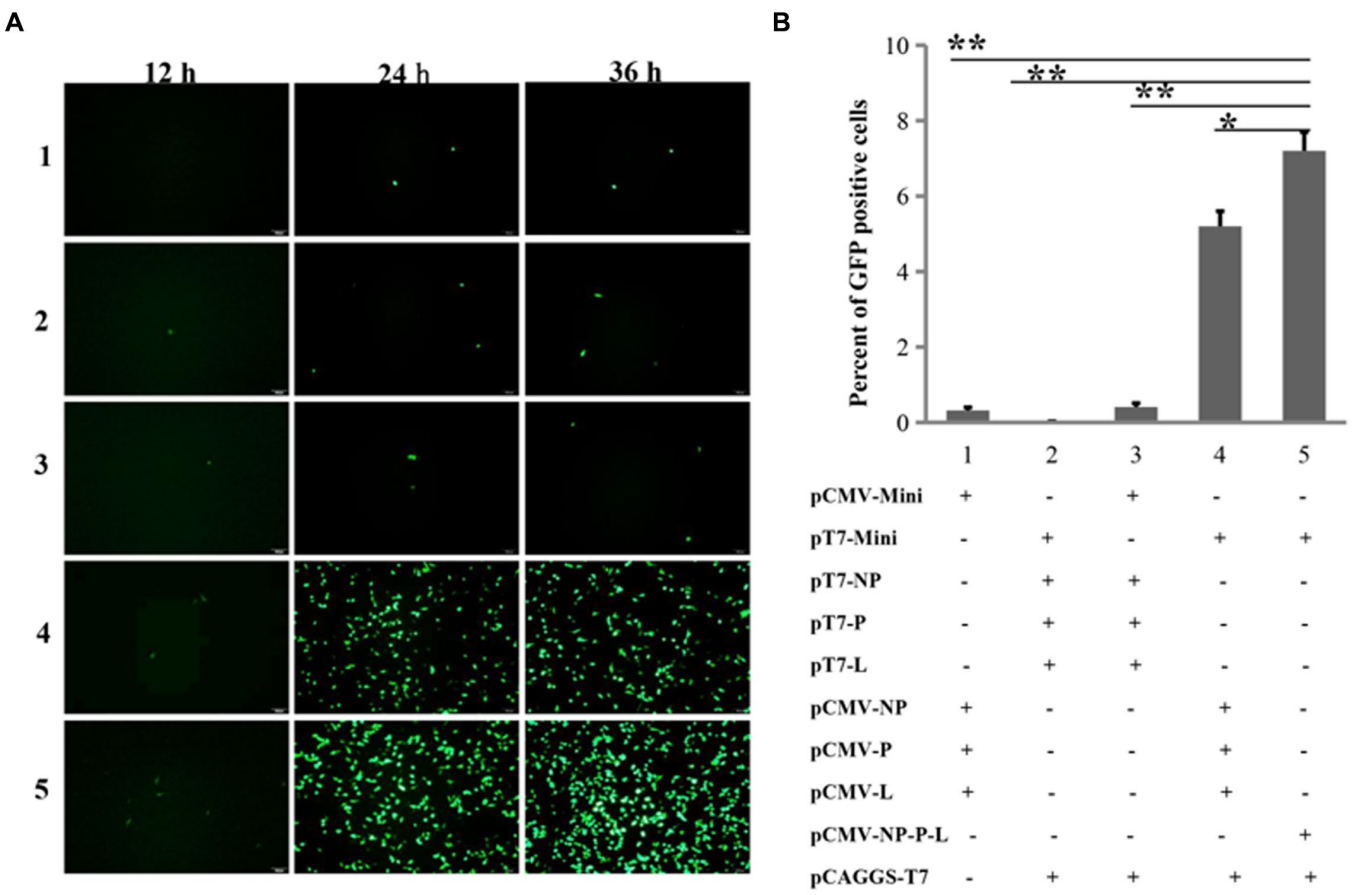
Comparing the efficiencies of NDV reverse genetics at the mini-genome level. **(A)** Fluorescent detection using a microscope. GFP-expressing cells were imaged every 12 h for 36 h post-transfection. **(B)** Fluorescent detection via flow cytometry. At the 24th h post-transfection, cells were harvested to assess the proportion of GFP-positive cells using flow cytometry. ^*^p < 0.05, ^**^p < 0.01.

Since the replication and transcription of the NDV genome occur in the cytoplasm, while the CMV promoter is recognized by eukaryotic RNA polymerase II in the nucleus, it leads us to suppose that this two-plasmid system may not be able to rescue NDV from Mini prep plasmids due to its promoter. Promoters such as T7, which function in the cytoplasm, could potentially address this issue. In addition to the NP, P, and L proteins, a mini-genome consisting of three parts has been frequently utilized for optimizing reverse genetics. These parts include: (1) the region of the NDV genome from the Leader to the start codon of NP; (2) the reported gene; and (3) the region from the stop codon of L to the Trailer. To test the effectiveness of five NDV reverse genetics strategies based on mini-genomes, we assessed the expression of the reporter gene (GFP) post-transfection. Slight GFP expression was observed in all four groups at the 12th h post-transfection, followed by a significant increase in GFP-positive cells at the 24th and 36th h across all groups. Groups 4 and 5 exhibited notably stronger green fluorescence compared to the other groups (Figure 2A). At the 24th h post-transfection, cells were collected for flow cytometry analysis of GFP expression. The percentage of GFP-positive cells in groups 4 and 5 was significantly higher than in the other groups, reaching around 5 to 7% in these two groups (Figure 2B). These findings suggest that cloning the viral genome and NP, P, and L genes under the T7 and CMV promoters, respectively, significantly enhances the efficiency of NDV reverse genetics. Moreover, the expression of NP, P, and L proteins from a single plasmid can further improve efficiency.

### 3.3 The T7 three-plasmid system rescued NDV using Mini prep plasmids

**Figure 3:**
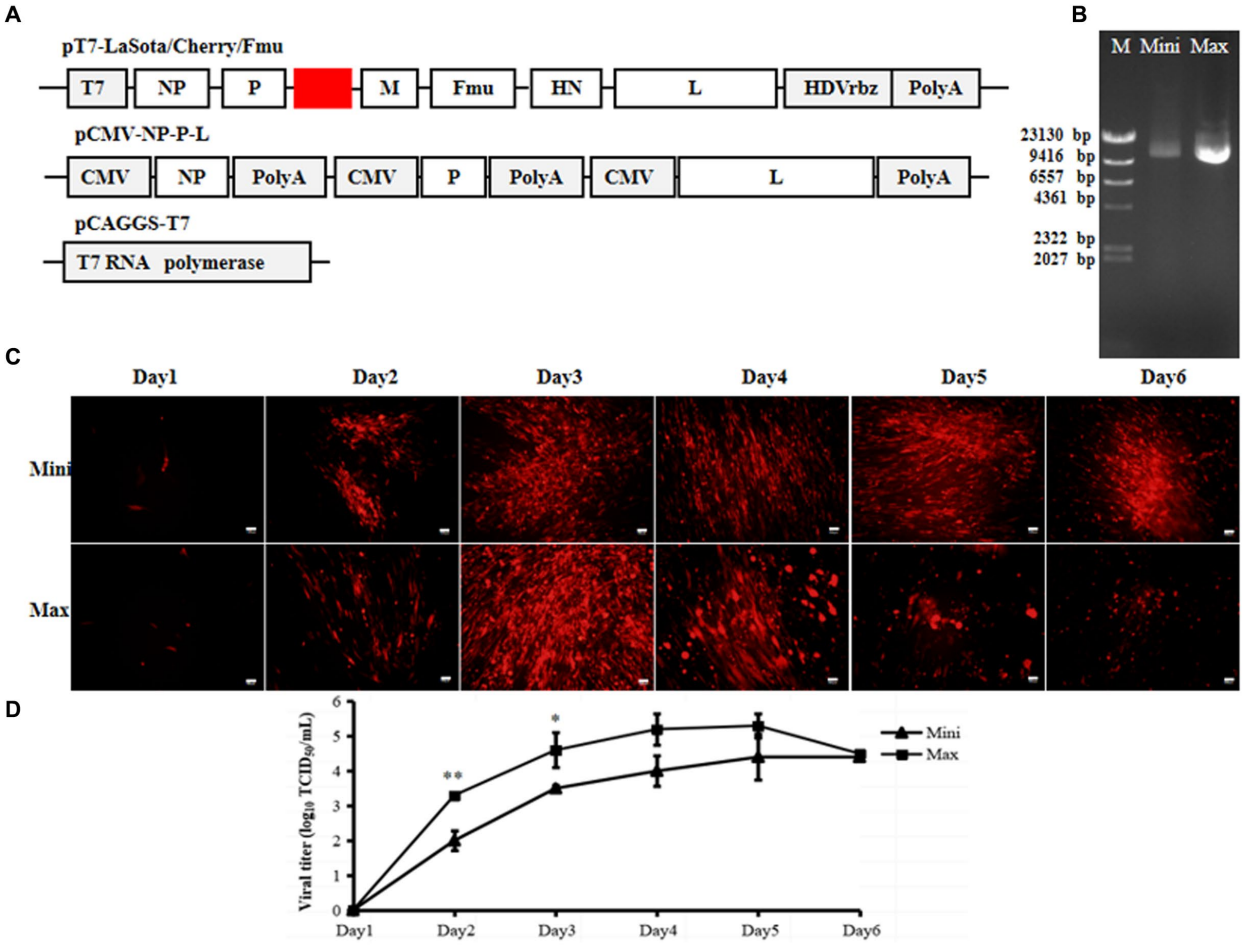
Efficiency of the 3-plasmid T7 NDV reverse genetics system. **(A)** Schematic diagrams of pT7-LaSota/Cherry/Fmu, pCMV-NP-P-L, and pCAGGS-T7 plasmids. **(B)** Mini prep and Maxi prep of pT7-LaSota/Cherry/Fmu. Electrophoresis was conducted on a 0.8% agarose gel with 1 μg of plasmids loaded. M, Mini, and Max represent Marker, Mini prep, and Maxi prep, respectively. **(C)** Fluorescent images of transfected cells. mCherry expression was monitored daily for 6 days post-transfection. **(D)** Viral titration in supernatants of transfected cells. Following transfection, 100 μL of supernatants from transfected cells were collected daily for 6 days and used to titrate rescued strains in BHK-21 cells. ^*^p < 0.05, ^**^p < 0.01.

Due to the full-length rLaSota/Cherry/Fmu cloned under the CMV promoter, we replaced the CMV promoter with the T7 promoter and HHrbz from the previous full-length plasmid, while keeping the rest of the parts unchanged (Figure 3A). This plasmid was prepared using both Mini and Maxi prep methods. Consistent with previous results, the quantity and quality of plasmids obtained through the Maxi prep method were significantly better than those obtained through the Mini prep method (Figure 3B). Subsequently, both plasmids containing pCMV-NP-P-L and pCAGGS-T7 were separately transfected into cells. Interestingly, one day post-transfection, red fluorescence was observed regardless of whether Mini or Maxi prep plasmids were used. Furthermore, increased RFP expression and CPE were observed in the transfected cells over time (Figure 3C). Meanwhile, supernatants were collected to determine the titer of rescued NDV. Viral particles were detected in all supernatants except on day 1 (Figure 3D). Viral titers increased gradually over time. For cells transfected with the Maxi prep plasmid, the virus titer reached up to 10^4.5^ TCID_50_/ml on day 4 post-transfection. Although viral titers in the supernatants of cells transfected with Mini prep plasmids were lower than those of cells transfected with Maxi prep plasmids during the first 3 days, the titer still reached around 10^4^ TCID_50_/ml towards the end of the transfection period (Figure 3D).

These results indicate that this T7 three-plasmid system can efficiently rescue velogenic-like NDV, even when the full-length plasmid is prepared using the Mini prep.

### 3.4 The T7 three-plasmid system also efficiently rescued lentogenic-like strains

**Figure 4:**
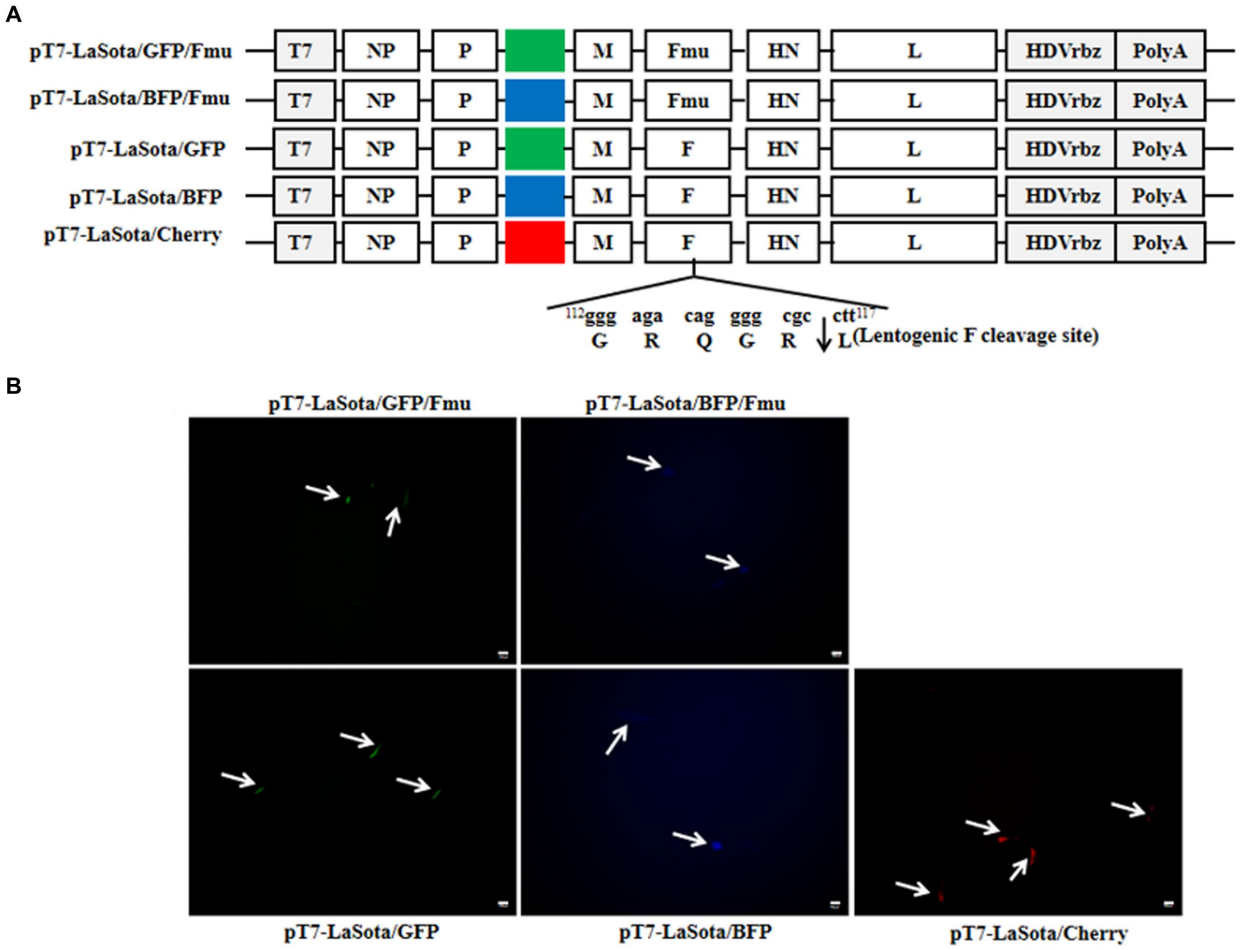
Rescue of NDV using the 3-plasmid T7 NDV reverse genetics system. **(A)** Diagrams of the other five full-length NDV plasmids. **(B)** Fluorescence in transfected cells. At the 1st d post-transfection, the full-length plasmids expressing fluorescent proteins were observed under the microscope. The white arrows indicate fluorescent cells.

**Figure 5:**
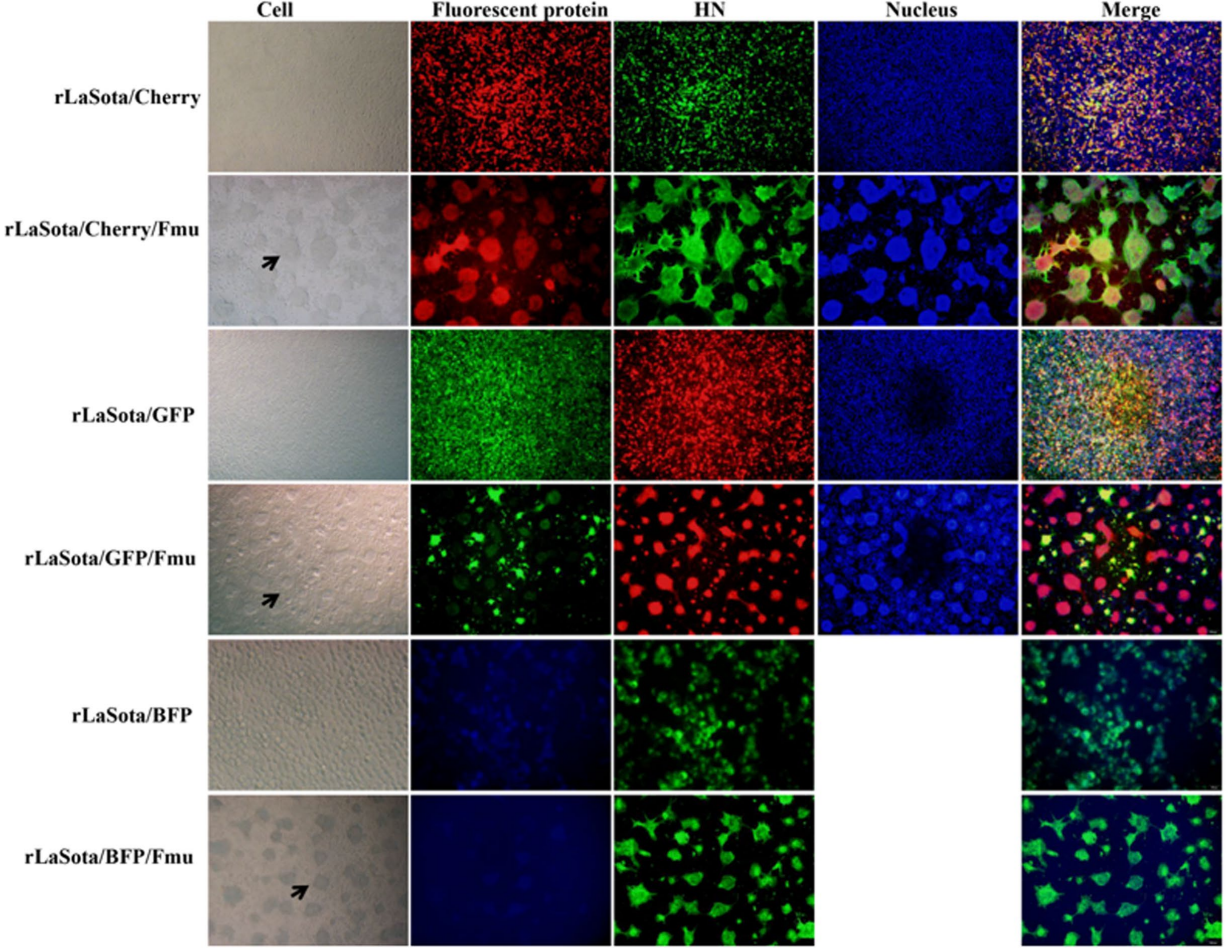
Confirmation of NDV strains by indirect immunofluorescence. The NDV HN antibody was utilized for IFA to verify the rescued strains. To detect rLaSota/Cherry, rLaSota/Cherry/Fmu, rLaSota/BFP, and rLaSota/BFP/Fmu, the secondary antibody used was goat anti-mouse Alexa Fluor 488, while for rLaSota/GFP and rLaSota/GFP/Fmu, it was goat anti-mouse Alexa Fluor 599. The black arrows indicate cell syncytia formed by infection with NDV strains containing the Velogenic F cleavage site.

The NDV with velogenic-type cleavage site of the NDV F protein can grow on transfected BHK-21 cells without trypsin-like proteases, making rescuing this type of NDV easier than that of lentogenic-like virus. To test whether the T7 three-plasmid system could efficiently generate more strains, especially lentogenic-like NDV, from Mini prep plasmids, five other full-length plasmids were constructed. Firstly, the mCherry gene of pT7-LaSota/Cherry/Fmu was replaced by GFP and BFP genes to obtain two additional full-genome plasmids. Subsequently, the cleavage site of the Fmu gene was modified to the lentogenic-like site to create three more full-genome plasmids (Figure 4A). All of these plasmids were purified using the Mini prep and then transfected along with pCMV-NP-P-L and pCAGGS-T7 into BHK-21 cells. Since all of these NDV full-genomes contained fluorescent genes, the fluorescence was not expressed in the absence of NP, P, and L proteins (data not shown). Regardless of whether the viral full-genomes had velogenic-like or lentogenic-like cleavage site, fluorescence was detected in all cells at the 24th h after transfection (Figure 4B). Three days post-transfection, cells with supernatants were collected and injected into 9-day-old chicken embryos for virus amplification. All rescued viruses were successfully amplified, as indicated by allantoic fluids with HA titers between 2^7^ and 2^9^ (data not shown). BHK-21 cells were infected with these six rescued fluorecent NDV strains. It was observed that fluorescent-positive cells also expressed the HN protein, suggesting that the fluorescence originated from the virus (Figure 5). Additionally, cells infected with strains containing the Fmu gene exhibited syncytia (Figure 5). These findings indicate that this modified reverse genetic system can efficiently rescue velogenic- and lentogenic-like NDV from Mini prep plasmids.

### 3.5 Recuing NDV strains with protein labeled tags

**Figure 6:**
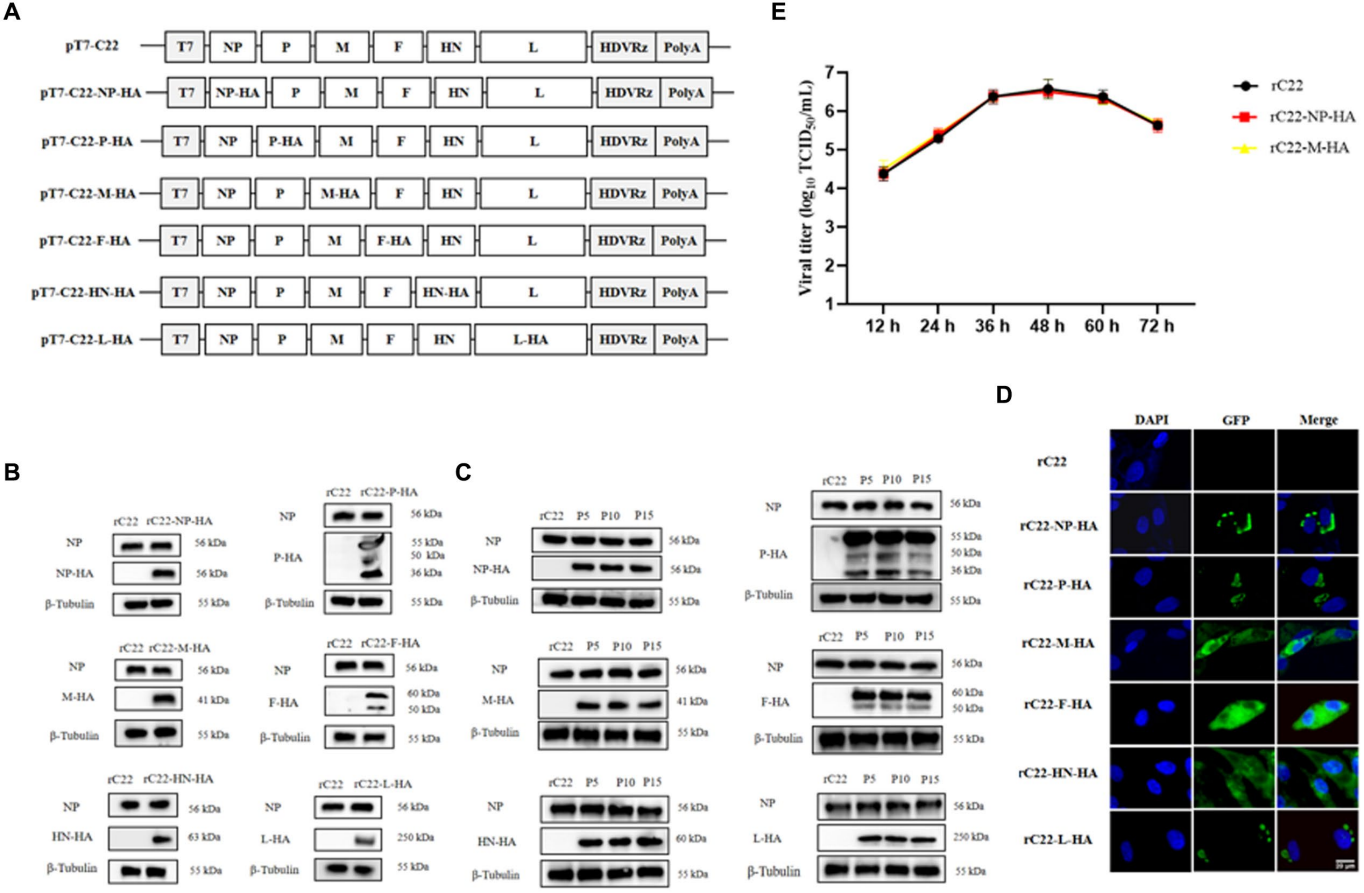
Construction and biological characterization of recombinant NDV virus with HA tag. **(A)** Diagrams depicting six full-length NDV C22 plasmids with integrated HA tags. **(B)** Detection of the viral protein fused with the HA tag via Western Blot using tag-specific antibodies. **(C)** Confirmation of the recombinant NDV virus harboring the HA tag through IFA using tag-specific antibodies. Infected cells were stained with the HA tag antibody (green) and DAPI (blue). **(D)** Stability assessment of the recombinant NDV virus. After 15 passages, each viral protein could still be detected using the HA tag antibody. **(E)** Evaluation of the growth capability of recombinant NDV rC22-NP-HA and rC22-M-HA.

**Figure 7:**
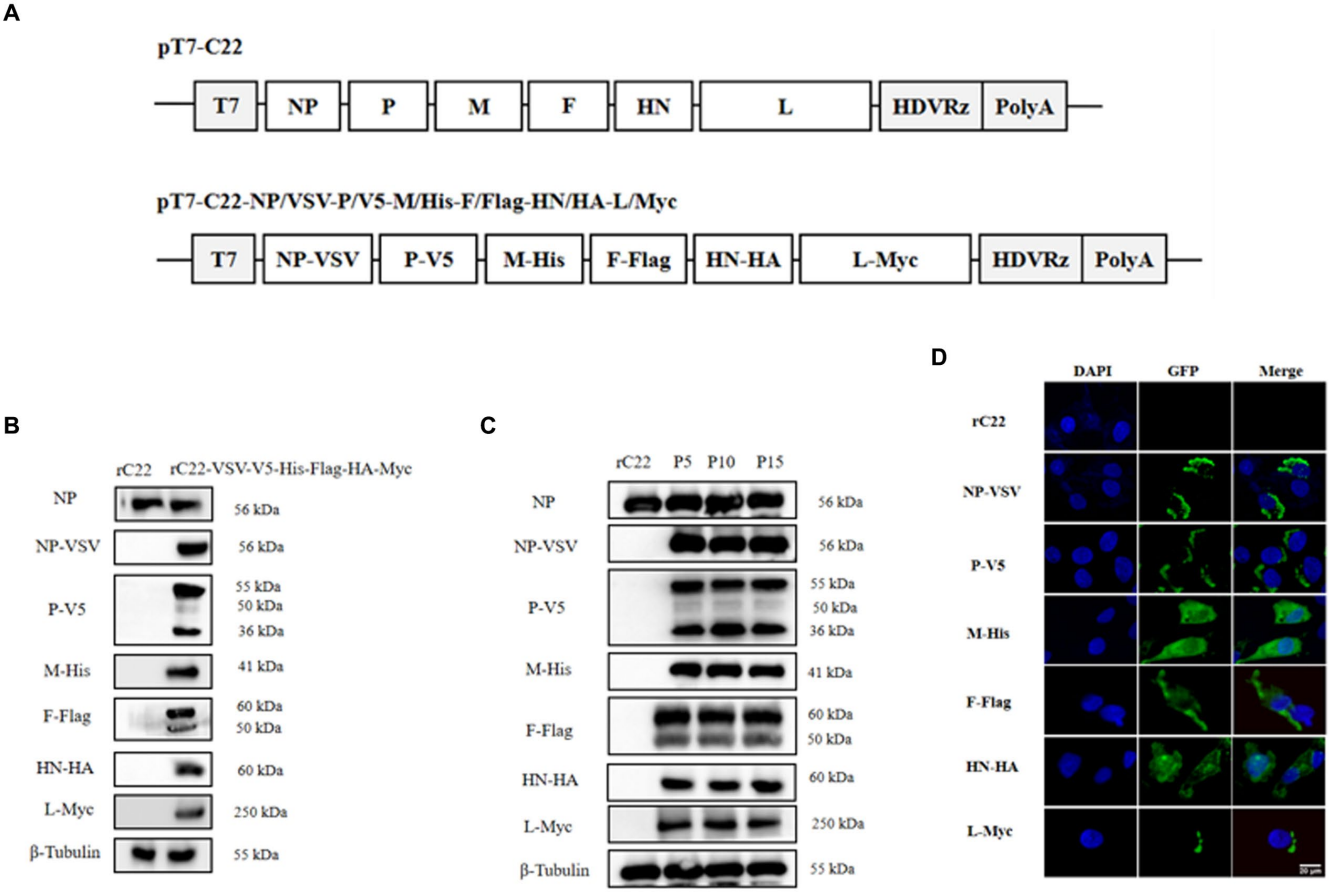
Construction and biological characterization of recombinant NDV virus with six tags. **(A)** Diagrams depicting full-length NDV C22 plasmids with integrated tags. **(B)** Detection of the viral protein fused with different tags via Western Blot using tag-specific antibodies. **(C)** Stability assessment of the recombinant NDV virus. After 15 passages, each viral protein could still be detected using tag antibody. **(D)** Confirmation of the recombinant NDV virus harboring tags through IFA using tag-specific antibodies. Infected cells were stained with the tag antibody (green) and DAPI (blue).

Based on this efficient reverse genetics approach, we successfully rescued six strains in which a single structural protein was fused with a HA tag (Figure 6A), as well as a strain in which every structural protein was labeled with a unique tag (Figure 7A). DF-1 cells were infected with these six rescued NDV strains to confirm the successful expression of the HA epitope tag. At the 24 hpi, total protein from DF-1 cells was extracted for Western blotting analysis. The results revealed that bands were identified using tag-specific antibodies and corresponded to the expected sizes of the NP, P, M, F, HN, and L proteins (Figures 6B, 7B). Even after 15 passages, the viral proteins could be detected with tag antibodies, while no specific protein could be detected in NDV without any tag (Figures 6C, 7C). Furthermore, using tag antibodies, the cleavage of the F protein could also be detected. Since the P protein and the unstructured V protein shared the same N-terminus and the tag-fused P protein was at the N-terminus, the V protein (36 kDa) was also identified in rC22-P-HA or rC22-VSV-V5-His-Flag-HA-Myc. Unexpectedly, a protein with a size around 50 kDa could be detected by tag antibodies in rC22-P-HA or rC22-VSV-V5-His-Flag-HA-Myc. Although the size of the L protein was up to 250 kDa, it could be easily detected by WB with tag antibodies in rC22-L-HA or rC22-VSV-V5-His-Flag-HA-Myc (Figures 6, 7).

Additionally, the cellular distribution of each NDV protein was checked using tag antibodies. The NP protein was clustered in the cytoplasm, the P protein showed a unipolar distribution in the cytoplasm, the M protein was located in both the nucleus and cytoplasm, the F and HN proteins were distributed in the cytoplasm, and the L protein was present in a unipolar distribution in the cytoplasm (Figures 6D, 7D). Subsequently, rC22-NP-HA and rC22-M-HA strains were chosen to test whether the tag could affect viral replication. We found that both strains showed similar growth ability as rC22 (Figure 6E). These results suggest that adding these small tags to the viral protein did not affect viral characteristics, such as infection, replication, protein expression and cellular location.

### 3.6 Screening of proteins interacting with the viral M protein during infection

**Figure 8:**
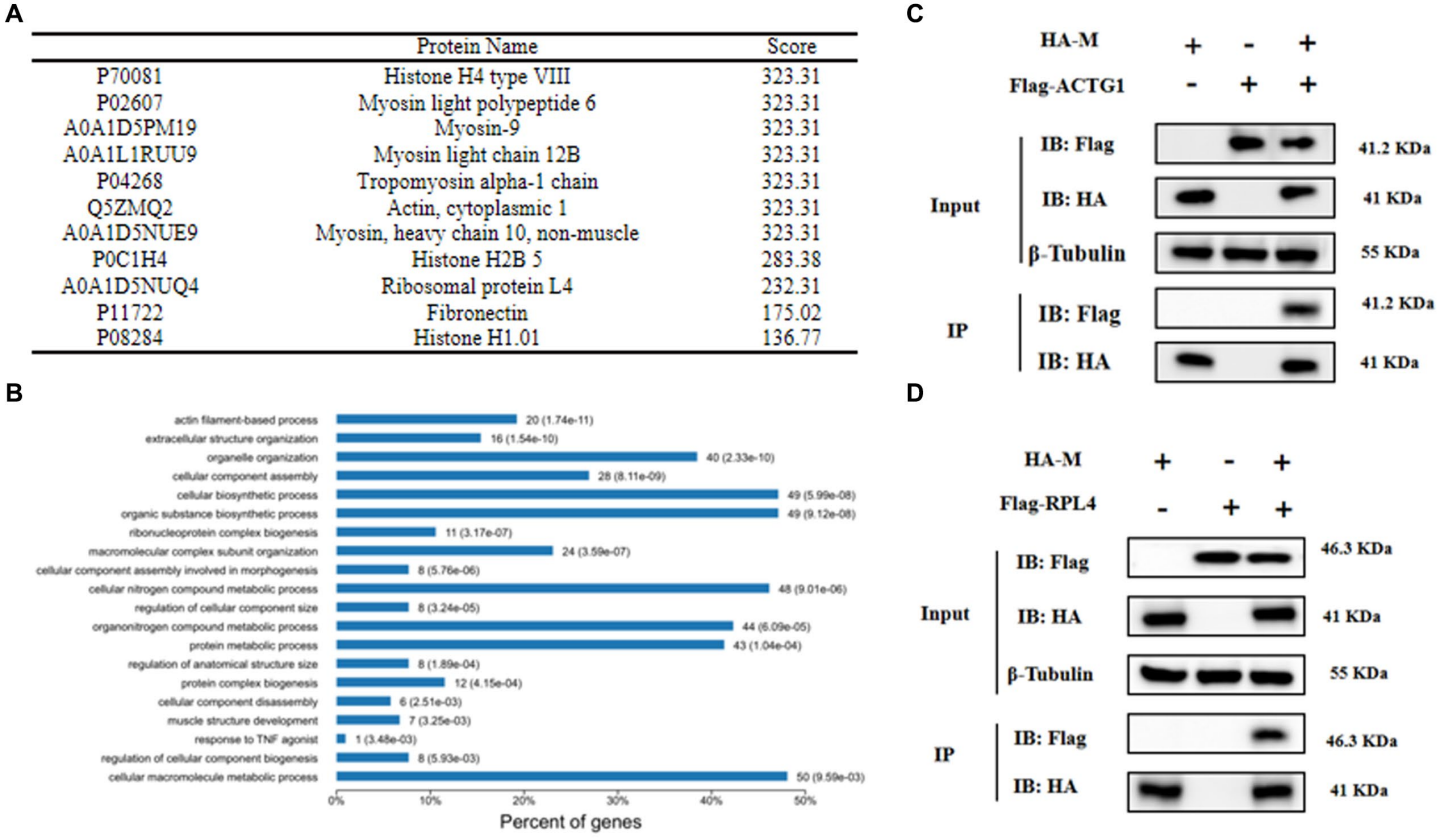
Analysis of proteins interacting with the M protein during infection. **(A)** Identification of the top 10 host proteins interacting with the M protein. **(B)** Biological processes of the interacting host proteins based on GO analysis. **(C,D)** Validation of the interaction between the viral M protein and chicken ACTG1 or RPL4. 293-T cells were co-transfected with plasmids expressing M and either ACTG1 or RPL4. After 48 h, cell protein samples were collected to confirm the interaction between the M protein and ACTG1 or RPL4 using Co-IP.

To assess the potential use of tag-labeled NDV strains for screening viral-host interactions, we utilized rC22-M-HA. Subsequent to infection, the interacting proteins were isolated through immunoprecipitation using HA tag antibody and identified via mass spectrometry. It was observed that M protein interacted with 100 host proteins. The potential M-interacting proteins are detailed in Supplementary Table S2. To verify the interaction between these identified proteins and the M protein, ACTG1 and RPL4 were chosen from the top 10 proteins based on scores (Figure 8A). The interaction of ACTG1 or RPL4 with the M protein was validated through Co-IP (Figures 8C,D). GO analysis was performed to investigate the functions of the interacting host proteins. The biological processes of these proteins mainly involved cellular macromolecule metabolic processes, cellular component assembly, and cellular nitrogen compound metabolic processes (Figure 8B). These findings suggest that tag-labeled NDV can serve as a valuable tool for comprehensively understanding virus-host interactions.

## 4 Discussion

By combining the two-plasmid system with the T7 promoter, a NDV reverse genetics system was developed in this study. This system efficiently rescued velogenic- and lentogenic-like NDV strains from Mini prep plasmids, eliminating the need for maxi-prep and potentially proving valuable for NDV research and optimizing reverse genetics of other NSNS viruses.

In contrast to rescuing positive-sense RNA viruses, rescuing NSNS viruses relies on the viral genome or anti-genome from plasmids transfected into cells (5). A higher quantity of full-genome plasmid increases the likelihood of obtaining correct full-length viral RNA after transfection. To generate sufficient full-length viral RNA, rescuing NSNS viruses requires a large quantity of plasmids, sometimes up to 10 μg (9, 17). Maxi prep plasmids exhibit superior quality and higher quantity compared to Mini prep, making NSNS virus rescuing reliant on plasmids purified by Maxi prep. However, Maxi prep is more costly, time-consuming, labor-intensive, and requires specialized equipment compared to Mini prep, rendering it inconvenient. Previously, we developed a two-plasmid system with the CMV promoter for rescuing NDV (9). This system aimed to decrease the number of plasmids used, potentially increase the number of cells co-expressing viral genome, NP, P, and L proteins, thereby enhancing rescuing efficiency. However, all rescues were still dependent on Maxi prep plasmids. Initially, we attempted to test whether this system could generate NDV using Mini prep plasmids. Unfortunately, this efficient system failed to rescue NDV from Mini prep plasmids but successfully worked with Maxi prep plasmids as shown in Figure 1, indicating that the quantity of plasmids can impact NDV rescuing. Since this system operated under the CMV promoter, which functions in the nucleus, it may not be suitable for rescuing viruses that replicate in the cytoplasm (18, 19). Subsequently, we replaced the CMV promoter of the full-length or mini-genome plasmid with the T7 promoter. As anticipated, this modification significantly increased the efficiency of the mini-genome (Figure 2) and successfully rescued velogenic- and lentogenic-like NDV from Mini prep plasmids (Figures 3–5). Additionally, fluorescence was detected in cells one day post-transfection with Mini prep plasmids, suggesting that NDV rescuing may only require one day in transfected cells (Figure 3). While the titer of the rescued strain based on Mini prep was lower than that of Maxi prep in the first three days post-transfection, it was higher than that of the transfection using the CMV promoter system with Maxi prep. These findings indicate that the rescuing efficiency of this modified system using Mini prep plasmids surpasses that of the previous two-plasmid system with Maxi prep plasmids.

Following fluorescence detection and viral titration, we observed that the growth capacity of rLaSota/Cherry/Fmu was not as robust as that of other velogenic fluorescent NDV strains rescued from genotype XI or VII (data not shown). However, it was still capable of replicating in the rescued cells (10, 20). Since NDV with velogenic-like fusion protein can amplify in rescued cells, rescuing this type of virus is efficient (13). On the contrary, rescuing NDV with lentogenic-like fusion proteins is challenging due to its inability to replicate in cells without trypsin supplementation (9, 21, 22). Nevertheless, candidates for attenuated ND vaccines or other NDV vector vaccines typically involve viruses with lentogenic-like fusion protein for reasons related to virulence and biosafety (23). Therefore, there is an urgent need for an efficient system to rescue NDV with lentogenic-like fusion proteins. In this study, utilizing the modified system, cells transfected with plasmids containing the full-length NDV genome with lentogenic-like F protein exhibited fluorescence one day after transfection. Furthermore, following amplification in chicken embryos, all NDV strains with lentogenic-like F protein were successfully obtained. Consequently, this newly developed reverse genetic system demonstrates the capability to efficiently rescue NDV regardless of the cleavage site of viral F protein.

Previous research has extensively documented the integration of the GFP tag into the NDV genome. GFP, a widely employed marker, has emerged as a valuable asset for monitoring viral infections (14). Apart from fluorescent tags, various other types of markers have been utilized across a spectrum of viruses. When these markers are applied to viruses, it is imperative to ensure that the size and placement of the inserted marker do not compromise the functionality of the target protein. While some markers are appended to viral proteins at their C or N-terminus (24), others may be accommodated within internal loops (14). Additionally, certain viral proteins can support larger markers such as GFP, protein A, and glutathione-S-transferase, while others are compatible with smaller markers like VSV, V5, His, Flag, HA, and Myc (25). Smaller markers are often favored due to their reduced size, which minimizes disruption to protein function, localization, and interactions.

Utilizing the efficient NDV reverse genetics system, we successfully incorporated HA epitope tags at the C-termini of NP, M, F, HN, and L proteins, as well as at the N-terminus of the P protein. This led to the creation of six recombinant NDV viruses- rC22-NP-HA, rC22-P-HA, rC22-M-HA, rC22-F-HA, rC22-HN-HA, and rC22-L-HA. Additionally, we engineered an NDV strain with six tags integrated. In this modified virus, NP, M, F, HN, and L proteins each carry VSV, His, Flag, HA, and Myc tags, respectively, at their C-termini, while the P protein is tagged with a V5 tag at its N-terminus. Subsequent to infection, these viruses effectively expressed the tags and exhibited genetic stability. By utilizing tag-specific antibodies, the tagged viral proteins could be readily identified, regardless of their size, cleavage, or modifications. Importantly, the insertion of tags into NDV proteins did not compromise the essential biological features of NDV, including infection, replication, protein expression, and localization. Consequently, these tagged NDV strains serve as a potent tool for investigating the interactions between NDV and its host.

Through coevolution with their hosts over time, viruses have developed intricate strategies to hijack cellular factors for viral uptake, replication, and transmission. Studies have demonstrated that the nuclear localization of paramyxovirus M protein (26) inhibits the transcription of host proteins. Although the transcription and replication of NDV take place in the cytoplasm, early in infection, viral M proteins are localized in the nucleus (27–30). This nuclear localization believed to impede the production of host proteins by altering essential nuclear components required for host transcript synthesis (31, 32). However, the specifics of NDV M protein interactions with host proteins remain unclear. In this investigation, we employed the tagged rC22-M-HA virus in combination with AP-MS to screen for host proteins that interact with the M protein during NDV infection of DF-1 cells. Our analysis revealed that M potentially interacted with 100 host proteins in addition to NDV NP, P, F, HN, and L proteins. Gene Ontology analysis suggested that these host proteins primarily comprised cytoskeletal proteins, ribosomal proteins, etc., indicating their potential involvement in NDV assembly and budding processes. KEGG pathway analysis highlighted the participation of host proteins in pathways such as Ribosome, Endocytosis, Phagosome, and Adherens junction.

The interactions of the M protein with host proteins Actin, cytoplasmic 1 (ACTG1) and large ribosomal protein 4 (RPL4) were validated by co-immunoprecipitation. ACTG1, a member of the γ-actin family, has been reported to interact with HIV-1 proteins, inhibiting viral assembly and production (33). The interaction of ACTG1 with paramyxoviruses had not been previously documented. The physical binding of NDV M proteins to actin was identified (34), suggesting a potential role of the M protein-ACTG1 interaction in NDV assembly and budding processes. RPL4 is a highly conserved ribosomal subunit involved in protein translation and ribosome assembly. Studies have shown that overexpression of RPL4 reduces retroviral virion assembly and release (35). During Epstein Barr Virus infection, upregulation of RPL4 expression and its nuclear relocalization facilitate the formation of a ternary complex with Nuclear Antigen 1 (EBNA1) and NCL, promoting persistent infection.

In addition to 100 host proteins, we also found that the M protein interacts with five NDV structural proteins, including NP, P, F, HN, and L proteins. Studies have proved that the NDV M protein directly interacts with the viral F and HN proteins, with the cytoplasmic tails of the F and HN protein complexes filling the gaps between the M protein dimers (36–38). It has also been shown that the M protein interacts with the RNP complex (39). Our results confirm the pivotal role of the M protein in guiding viral assembly through its interactions with other viral proteins.

## 5 Conclusion

In this study, NDV strains carrying tagged structural proteins were rescued. These tags not only facilitated the easy detection of viral proteins but also had no discernible impact on viral replication, genetic stability, protein expression and protein cellular localization. Utilizing tag-specific antibodies, numerous host proteins potentially interacting with the viral M protein were screened, laying the groundwork for further research on virus-host interactions.

## Data availability statement

The datasets presented in this study can be found in online repositories. The names of the repository/repositories and accession number(s) can be found in the article/Supplementary material.

## Ethics statement

The manuscript presents research on animals that do not require ethical approval for their study.

## Author contributions

RW: Data curation, Validation, Writing – original draft. XC: Investigation, Writing – original draft. KL: Investigation, Writing – original draft. ZC: Formal analysis, Writing – original draft. XD: Resources, Writing – original draft. HG: Investigation, Writing – original draft. XW: Software, Writing – original draft. RD: Software, Writing – original draft. JW: Data curation, Writing – original draft. XW: Formal analysis, Writing – original draft. SX: Writing – original draft, Methodology. HL: Funding acquisition, Project administration, Writing – review & editing. ZY: Project administration, Writing – review & editing, Funding acquisition, Resources.
