# Supplementary material for: Rescuing Newcastle disease virus with tag for screening viral-host interacting proteins based on highly efficient reverse genetics
Source: Front Vet Sci. 2024 Jul 19;11:1418760. doi: 10.3389/fvets.2024.1418760 (PMC11294249; doi:10.3389/fvets.2024.1418760)
Supplement: Supplementary file 1 [file Table_1.DOCX]

| Plasmids | Properties |
| --- | --- |
| pCMV-Mini | Minigenome, under CMV promoter, consists of Leader, Trailer of LaSota and EGFP of pEGFP-C1. |
| pT7-Mini | Minigenome, under T7 promoter, consists of Leader, Trailer of LaSota and EGFP of pEGFP-C1. |
| pT7-NP | Expressing NP protein of LaSota under T7 promoter. |
| pT7-P | Expressing P protein of LaSota under T7 promoter. |
| pT7-L | Expressing L protein of LaSota under T7 promoter. |
| pCMV-NP | Expressing NP protein of LaSota under CMV promoter. |
| pCMV-P | Expressing P protein of LaSota under CMV promoter. |
| pCMV-L | Expressing L protein of LaSota under CMV promoter. |
| pCMV-NP-P-L | Expressing NP, P and L protein of LaSota with individual CMV promoter. |
| pCAGGS-T7 | Expressing T7 RNA polymerase from pCAGGS vector. |
| pCMV-LaSota/Cherry/Fmu | The full genome of LaSota strain was under CMV promoter. The mCherry gene was inserted between P and M genes. Cleavage site of F protein belonged to velogenic type. |
| pT7-LaSota/Cherry/Fmu | The full genome of LaSota strain was under T7 promoter. The mCherry gene was inserted between P and M genes. Cleavage site of F protein belonged to velogenic type. |
| pT7-LaSota/GFP/Fmu | The full genome of LaSota strain was under T7 promoter. The EGFP gene was inserted between P and M genes. Cleavage site of F protein belonged to velogenic type. |
| pT7-LaSota/BFP/Fmu | The full genome of LaSota strain was under T7 promoter. The BFP gene was inserted between P and M genes. Cleavage site of F protein belonged to velogenic type. |
| pT7-LaSota/Cherry | The full genome of LaSota strain was under T7 promoter. The mCherry gene was inserted between P and M genes. Cleavage site of F protein belonged to lentogenic type. |
| pT7-LaSota/GFP | The full genome of LaSota strain was under T7 promoter. The EGFP gene was inserted between P and M genes. Cleavage site of F protein belonged to lentogenic type. |
| pT7-LaSota/BFP | The full genome of LaSota strain was under T7 promoter. The BFP gene was inserted between P and M genes. Cleavage site of F protein belonged to lentogenic type. |
| pT7-C22 | The full genome of NDV Genotype VII C22 strain under T7 promoter. |
| pT7-C22-NP-HA | The full genome of the modified C22 strain, in which the NP protein was fused with HA tag. |
| pT7-C22-P-HA | The full genome of modified C22 strain, in which the P protein was fused with HA tag. |
| pT7-C22-M-HA | The full genome of modified C22 strain, in which the M protein was fused with HA tag. |
| pT7-C22-F-HA | The full genome of modified C22 strain, in which the F protein was fused with HA tag. |
| pT7-C22-HN-HA | The full genome of modified C22 strain, in which the HN protein was fused with HA tag. |
| pT7-C22-L-HA | The full genome of modified C22 strain, in which the L protein was fused with HA tag. |
| pT7-C22-NP/VSV-P/V5-M/His-F/Flag-HN/HA-L/Myc | The full genome of modified C22 strain, in which the NP, P, M, F, HN and L protein was fused with VSV, V5, His, Flag, HA and Myc tag, respectively. |
| pCAGGS-M-HA | Expressing viral M protein with HA tag from pCAGGS vector |
| pCAGGS-ACTG1-Flag | Expressing chicken ACTG1 protein with Flag tag from pCAGGS vector |
| pCAGGS-RPL4-Flag | Expressing chicken RPL4 protein with Flag tag from pCAGGS vector |

**TableS1. Information of Plasmids used in this study**
