# Supplementary material for: Rescuing Newcastle disease virus with tag for screening viral-host interacting proteins based on highly efficient reverse genetics
Source: Front Vet Sci. 2024 Jul 19;11:1418760. doi: 10.3389/fvets.2024.1418760 (PMC11294249; doi:10.3389/fvets.2024.1418760)
Supplement: Supplementary file 2 [file Table_2.DOCX]

**Table S2. Host proteins interacting with M protein**

| Protein ID | Protein Name | Score |
| --- | --- | --- |
| P70081 | Histone H4 type VIII | 323.31 |
| P02607 | Myosin light polypeptide 6 | 323.31 |
| A0A1D5PM19 | Myosin-9 | 323.31 |
| A0A1L1RUU9 | Myosin light chain 12B | 323.31 |
| P04268 | Tropomyosin alpha-1 chain | 323.31 |
| Q5ZMQ2 | Actin, cytoplasmic 1 | 323.31 |
| A0A1D5NUE9 | Myosin, heavy chain 10, non-muscle | 323.31 |
| P0C1H4 | Histone H2B 5 | 283.38 |
| A0A1D5NUQ4 | Ribosomal protein L4 | 232.31 |
| P11722 | Fibronectin | 175.02 |
| P08284 | Histone H1.01 | 136.77 |
| P84229 | Histone H3.2 | 132.25 |
| A0A3Q2UDG5 | Myosin XVIIIA | 131.98 |
| A0A1L1RL76 | 60S ribosomal protein L14 | 130.12 |
| A0A1D5PT58 | 40S ribosomal protein S8 | 128.92 |
| F1NJ08 | Vimentin | 101.29 |
| A0A452J805 | Tropomyosin beta chain | 97.972 |
| P28497 | F-actin-capping protein subunit alpha-2 | 94.455 |
| P00356 | Glyceraldehyde-3-phosphate dehydrogenase | 91.448 |
| A0A1D5PCT4 | Coronin | 82.773 |
| A0A1D5NZ30 | Nucleolin | 79.6 |
| P60706 | Actin, cytoplasmic 1 | 75.102 |
| A0A1D5PAF9 | AP-3 complex subunit beta | 68.247 |
| P18302 | Drebrin | 64.857 |
| P07583 | Beta-galactoside-binding lectin | 62.602 |
| P63270 | Actin, gamma-enteric smooth muscle | 62.061 |
| Q8UWG7 | 60S ribosomal protein L6 | 60.968 |
| P32429 | 60S ribosomal protein L7a | 52.582 |
| P13127 | F-actin-capping protein subunit alpha-1 | 47.775 |
| E1C2F2 | Pinin | 42.677 |
| Q98TF7 | 60S ribosomal protein L35 | 39.468 |
| A0A1L1S0X5 | 60S ribosomal protein L34 | 39.088 |
| F1NIX0 | 60S ribosomal protein L8 | 38.106 |
| Q98TF8 | 60S ribosomal protein L22 | 36.149 |
| F1NK75 | Tropomyosin 4 | 35.738 |
| A0A1D5PMQ5 | Keratin, type I cytoskeletal 42-like | 33.756 |
| A0A1D5NUI1 | Ribosomal protein L3 | 30.51 |
| H9L074 | Tropomyosin 3 | 29.57 |
| F1NPD3 | 60S ribosomal protein L18a | 26.383 |
| A0A1L1RLL0 | Ribosomal protein L24 | 25.903 |
| Z4YJB8 | Destrin | 25.775 |
| Q90WD0 | Actin-related protein 3 | 25.718 |
| F6SU35 | Ribosomal protein | 25.223 |
| A0A3Q2TZ02 | Myosin ID | 24.901 |
| F1NRG3 | Treacle ribosome biogenesis factor 1 | 24.885 |
| F1NQ35 | 60S ribosomal protein L35a | 23.893 |
| A0A1D5NXY4 | Barrier to autointegration factor 1 | 23.388 |
| P47838 | 40S ribosomal protein S6 | 22.117 |
| F1NTJ5 | Myosin IB | 20.916 |
| F1NQG5 | Ribosomal protein L15 | 20.036 |
| Q5ZKK8 | Ribosomal protein L19 | 19.938 |
| P67883 | 60S ribosomal protein L30 | 19.77 |
| Q5ZJ56 | 60S ribosomal protein L7 | 18.898 |
| Q6W8X3 | High mobility group A1b | 18.417 |
| A0A1D5PD89 | Natural killer cell triggering receptor | 17.679 |
| A0A1D5PMT8 | 60S acidic ribosomal protein P2 | 16.829 |
| F1NN16 | 40S ribosomal protein S7 | 16.591 |
| Q90835 | Elongation factor 1-alpha 1 | 16.401 |
| P41125 | 60S ribosomal protein L13 | 16.324 |
| Q7ZTG3 | ARF tumor suppressor | 13.919 |
| A0A3Q3ANM5 | Actin-related protein 2/3 complex subunit 4 | 13.618 |
| A0A1L1RT94 | 60S ribosomal protein L31 | 13.42 |
| O73885 | Heat shock cognate 71 kDa protein; | 13.374 |
| F1NBX4 | 60S ribosomal protein L27a | 13.215 |
| P21566 | Cofilin-2 | 11.669 |
| P47836 | 40S ribosomal protein S4 | 11.659 |
| Q02440 | Unconventional myosin-Va | 11.634 |
| Q5ZLY3 | Tropomodulin 3 | 11.547 |
| P18660 | 60S acidic ribosomal protein P1 | 10.791 |
| P79781 | Ubiquitin-40S ribosomal protein S27a | 8.6531 |
| E1C1G5 | Zinc finger CCCH-type containing 18 | 8.4328 |
| Q5ZHW8 | Ribosomal protein S14 | 8.1154 |
| A0A1D5PKI8 | Glypican 4 | 7.6807 |
| A0A1D5P3B1 | 60S ribosomal protein L11 | 7.4354 |
| Q08200 | 60S ribosomal protein L10 (Fragment) | 7.4297 |
| F6R7D0 | Histone deacetylase complex subunit SAP18 | 7.0834 |
| F1NIK4 | Ribosomal protein L36a | 7.0248 |
| A0A1L1RNZ2 | 60S ribosomal protein L29 | 7.0233 |
| E1BQC5 | Poly [ADP-ribose] polymerase | 6.7896 |
| A0A3Q2TU46 | Exocyst complex component | 6.7896 |
| A0A3Q2TV39 | 40S ribosomal protein S27 | 6.7643 |
| P14315 | F-actin-capping protein subunit beta isoforms 1 and 2 | 6.6676 |
| P47832 | 60S ribosomal protein L26 (Fragment) | 6.6429 |
| F1NYR0 | HYDIN, axonemal central pair apparatus protein | 6.5522 |
| F1NHB9 | TATA element modulatory factor 1 | 6.4401 |
| F1NZH0 | SLIT and NTRK like family member 1 | 6.3662 |
| A0A1D5NU03 | ST3 beta-galactoside alpha-2,3-sialyltransferase 3 | 6.366 |
| A0A1D5NTD3 | Proteasomal ubiquitin receptor ADRM1 | 6.2405 |
| Q5ZMD6 | Histone H2A.Z | 6.2188 |
| Q98TH5 | 40S ribosomal protein S11 | 6.1643 |
| A0FKN5 | Prestin | 6.1339 |
| R4GKR1 | Interleukin 5 receptor subunit alpha | 6.0205 |
| P61355 | 60S ribosomal protein L27 | 5.9446 |
| F1NPA9 | DNA-(apurinic or apyrimidinic site) lyase | 5.9439 |
| F1P413 | Mediator of RNA polymerase II transcription subunit 13 | 5.8939 |
| Q98TF6 | 60S ribosomal protein L36 | 5.8697 |
| A0A1L1RZJ6 | ATP synthase subunit alpha | 5.8628 |
| E1BQW9 | CST complex subunit STN1 | 5.8516 |
| E1C4C7 | Replication protein A3 | 5.8314 |
| A0A1D5P4V9 | 40S ribosomal protein S24 | 5.8083 |
